# Examination within a Photonic Memory-Based Framework: Al and In Dual-Doped ZnO Thin Film UV Photosensor Devices

**DOI:** 10.1021/acsami.6c01334

**Published:** 2026-03-30

**Authors:** Emre Kartal, Osman Kahveci, Abdullah Akkaya, Mücahit Yılmaz, Raşit Aydın, Bünyamin Şahin

**Affiliations:** a Department of Physics, Faculty of Sciences, 52958 Erciyes University, 38039 Kayseri, Turkiye; b Erciyes University METALION Research Group, 38039 Kayseri, Turkiye; c Energy Conversion Research and Application Center, Erciyes University, 38039 Kayseri, Turkiye; d Mucur Technical Vocational Schools, Technical Progress Department, Kırşehir Ahi Evran University, 40100 Kırşehir, Turkiye; e Department of Basic Sciences, Faculty of Engineering, 539861 Necmettin Erbakan University, 42090 Konya, Turkiye; f Department of Physics, Faculty of Sciences, 52993 Selcuk University, 42250 Konya, Turkiye

**Keywords:** ZnO thin films, photonic memory, photosensing, semiderivative

## Abstract

The main focus of our study is to examine and report the room-temperature enhancement of the optoelectronic and photoresponsive performance of ZnO materials induced by dual doping with Al and In, achieved by tailoring the materials’ structural and morphological characteristics. Structural analyses confirmed the formation of the hexagonal wurtzite ZnO phase, while doping induced noticeable modifications in the microstructure and morphology of the films. The crystallite size was found to decrease from 54.96 nm for undoped ZnO to 38.66 nm for ZnO/1.0% Al/1.0% In films, indicating dopant-induced grain refinement. Also, morphological analysis revealed that the particle thickness decreased from ∼400.1 to ∼201.6 nm, resulting in an increased surface:volume ratio. We conducted a systematic investigation focusing primarily on their persistent photoconductivity characteristics. Further analyses were performed to examine the detailed mechanisms of photocurrent generation, to observe photonic memory effects, to determine photosensor properties and device sensitivity, and to assess the effects of traps. The sensor devices were tested under a UV light source with a wavelength of 400 nm and an intensity of 75 μW/cm^2^, within the range of −5 to 5 V. In a dark environment, the current of the undoped ZnO film was 4.35 × 10^–6^ A, while in the 1.0% Al/1.0% In dual-doped device, this value increased approximately 11 times under UV illumination, reaching 2.11 × 10^–4^ A and yielding the highest photocurrent. Fast decay component τ_1_ showed similarity across all samples, varying between 24.74 and 35.68 s. The slow decay time (τ_2_) determining the memory capacity of the devices was measured as 302.89 s for undoped ZnO, while in the 1.0% Al/1.0% In doped sample, this time constant increased to 533.31 s, reaching its highest value. Our findings provide a basis for designing advanced photoactive materials for use in optoelectronic applications. They also indicate the optimal doping ratio for ZnO doped with 1.0% Al and In, supporting its potential use in photonic memory applications, such as multilevel data storage and optical programming.

## Graphical abstract

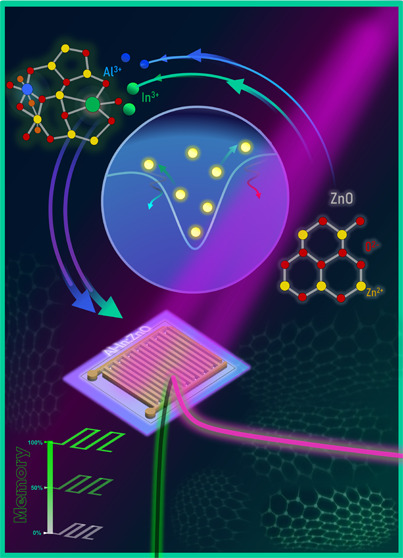

## 1 Introduction

Zinc oxide (ZnO) is one of the most extensively investigated metal oxide materials for scientific and technological applications^1,2^ because it is a wide band gap semiconductor (∼3.37 eV), has a high exciton binding energy (∼60 meV), and is chemically stable and practical with low-cost production methods.^3,4^ These properties make ZnO a promising material for a broad range of applications, including ultraviolet (UV) photodetectors, sensors, transparent electronics, and optoelectronic devices.^4–6^ Tailoring the electrical and optical performance of ZnO via doping with various metals enables controlled modification of carrier concentration, defect states, and the band structure.^6,7^ In particular, the photoresponse of ZnO-based materials has been extensively investigated due to their strong interaction with UV radiation and defect-sensitive charge-transport characteristics.^6,8,9^

Identifying the source of the photocurrent in semiconductors is essential for device fabrication. The primary sources of this photocurrent can be summarized as follows: absorption of incident light, excitation of electron–hole pairs, and transport of charge carriers. The total number of generated charge carriers, their recombination efficiency, and their mobility determine the conductivity of a semiconductor.^10^ ZnO-based materials exhibit a wide range of photoinduced electrical and optical phenomena, including tunable electrical conductivity, photoconductivity, and persistent photoconductivity. These properties are highly responsive to not only structural characteristics such as defect states, defect density, and surface chemistry but also external stimuli such as light, temperature, humidity, and the ambient atmosphere of ZnO.^9,11^ In addition, manufacturing methods or postproduction treatments have a significant impact on device performance and capabilities.^2^

One of the most interesting photoresponse phenomena observed in ZnO is persistent photoconductivity (PPC), defined as the long-term enhancement of electrical conductivity persisting after the light source is removed.^12,13^ In particular, PPC, characterized by the persistence of photoexcitation effects after the cessation of incident light, has attracted a significant amount of interest in the field of photonic devices.^13^ PPC is an undesirable feature for fast-switching devices, but it is highly desirable for some applications, such as optical memory elements, neuromorphic devices, and high-sensitivity photodetectors.^13^ The origin of PPC in ZnO is generally associated with defect-related carrier trapping and detrapping processes, surface adsorption and desorption of oxygen species, and, owing to the semiconductor nature of ZnO, the presence of deep-level states in the band gap.^12–16^ The relative contributions of each mechanism to PPC strongly depend on the synthesis method, microstructure, surface morphology, and impurity/doping content of the material.^16–18^ This dependence on carrier dynamics clearly leads to a wide variation in reported photoresponse behaviors under different illumination and environmental conditions.^12,13,15–18^

Doping is a widely used strategy to control the electrical and optical properties of ZnO by modulating its carrier concentration via defect and trap formation mechanisms.^2,19,20^ Group III elements such as aluminum (Al) and indium (In) are commonly used as dopants in ZnO to produce high-performance transparent conductive oxide films for solar cells, photodetectors, optoelectronic devices, and transparent electrodes.^2,21^ These donor dopants substitute for Zn^2+^ sites, causing n-type conductivity; as a consequence, the free-carrier density increases.^2^ These dopants also affect intrinsic defect formation mechanisms, lattice distortion, and surface states, which affect the PPC behavior of ZnO.^15,17,20^

The PPC phenomenon in ZnO is primarily correlated with intrinsic microstructural features, defects, and electronic structure.^12,15,17,22^ Thus, defect states and surface-mediated processes can lead to noticeable variations in crystallinity, morphology, and electronic structure, influencing carrier dynamics. Therefore, a combined analysis of structural, morphological, and optical properties is essential for reliably correlating the photoconductivity with defect-related electronic transitions and surface adsorption phenomena.

Djelloul et al.^23^ investigated undoped, Al-doped, and codoped (Al and In) ZnO thin films that were deposited by ultrasonic spray pyrolysis for solar cell applications. The lowest resistivity and highest carrier concentration were exhibited by codoped (Al and In) ZnO, due to the substitution of Zn^2+^ by trivalent Al^3+^ and In^3+^ ions, which supplied additional free electrons. The study concludes that Al- and In-doped ZnO thin films are the most promising material for use in electronic devices due to their good crystallinity and low resistivity.^23^

In the present study, we synthesized undoped, Al-doped, In-doped, and dual-doped (Al and In) ZnO samples using the SILAR method. These samples were systematically investigated, primarily focusing on their persistent photoconductivity characteristics. Finally, the role of the dopant type in PPC behavior and defect-mediated carrier dynamics was clarified using structural and optical characterization results. A comparative analysis was performed to reveal the interactions among dopant-induced defects, surface states, and photocarriers in doped and undoped ZnO-based films. Our findings provide a basis for designing advanced photoactive materials for optoelectronic applications.

## 2 Experimental Section

Undoped and doped ZnO thin films were synthesized using the successive ionic layer adsorption and reaction (SILAR) method. We opted for the SILAR method due to its simplicity and versatility, which enable researchers to experiment with novel combinations of thin films. We believe that our fabrication method can be adopted without the need for expensive equipment or facilities. Furthermore, our preliminary sensing performance results are promising and demonstrate the value of SILAR for research purposes.

All chemicals used in the study were of analytical grade and used without further purification. Zinc acetate dihydrate (C_2_H_6_O_3_Zn, Sigma-Aldrich, 99.999% trace metal basis), aluminum nitrate nonahydrate (AlH_18_N_3_O_18_, Sigma-Aldrich, 99.99% trace metal basis), and indium­(III) chloride (InCl_3_, Alfa Aesar, 99.99% metal basis) were used as starting salts. Each solution was prepared at a concentration of 0.1 M by dissolving the relevant salts in 100 mL of deionized water. The pH of the solutions was adjusted using ammonium hydroxide (NH_4_OH), thus ensuring that film formation occurred smoothly and consistently. The pH of the initial solution has a direct effect on the structural, morphological, optical, and sensing characteristics of the thin films. It plays a significant role because it impacts both adsorption and surface charge properties. For this reason, we determined that the most suitable value was approximately 10. This pH is controlled using an MRC (INE-PHS-3E), which displays the exact pH digitally.

During the experiment, the solution temperature was kept constant at 90 °C. Six film series were prepared: undoped ZnO, ZnO/1.0% Al, ZnO/1.0% In, ZnO/1.0% Al/1.0% In, ZnO/2.0% Al/1.0% In, and ZnO/1.0% Al/2.0% In. Each film was coated by using 15 SILAR cycles. A SILAR cycle consists of (i) immersing the substrate in the solution and (ii) rinsing with deionized water to remove loosely bound ions. The contribution ratios of Al and In were precisely controlled by adjusting the concentrations of the corresponding Al­(NO_3_)_3_·9H_2_O and InCl_3_ solutions. Upon completion of the coating process, the films were dried in ambient air and then annealed in an oven at 250 °C for 45 min to enhance surface adhesion and promote crystallinity. This value was chosen based on our previous study of the influence of annealing temperature.^24,25^ A schematic representation of the SILAR-based thin film growth process, including solution preparation, immersion–rinsing cycles, postcoating annealing stages, interdigitated electrode (IDE) preparation, and optoelectronic characterization of ZnO-based photosensor devices, is presented in Figure 1.

**Figure 1:**
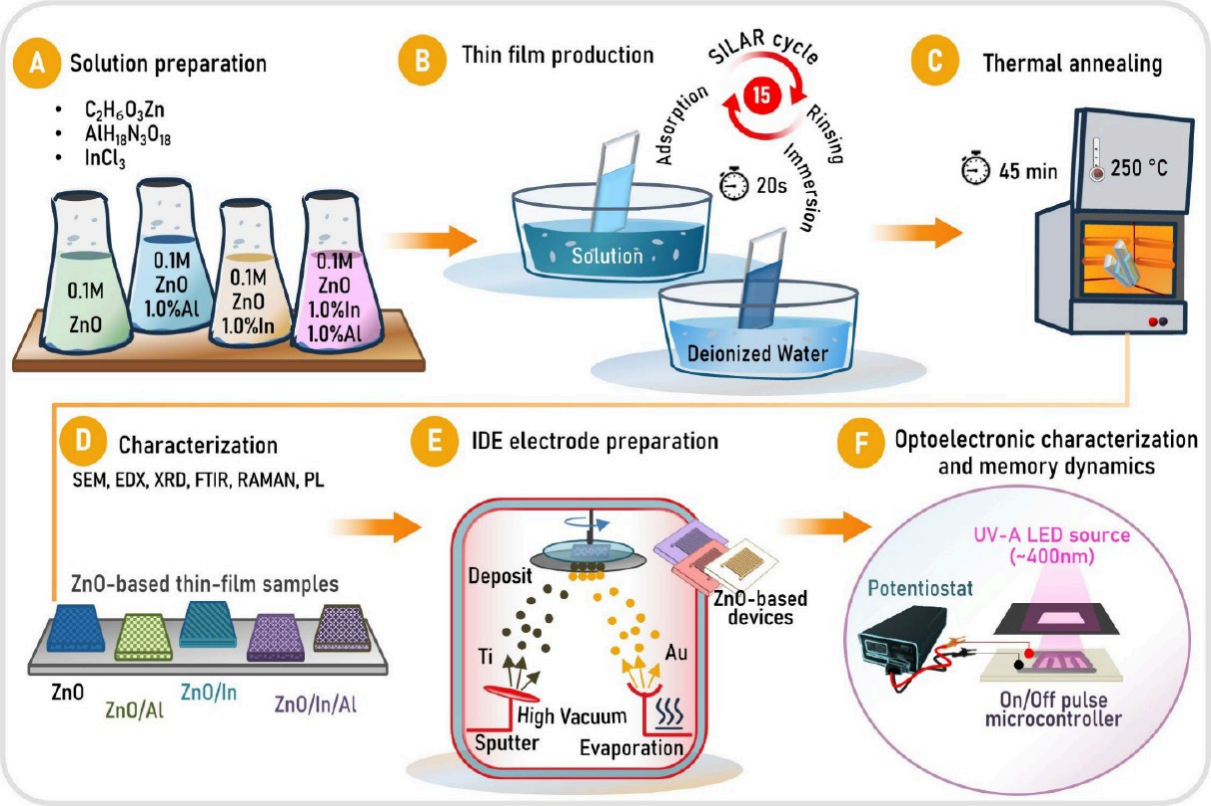
Schematic representation of SILAR-based thin film growth, IDE electrode preparation, and optoelectronic characterization processes for ZnO-based photosensor devices.

The synthesized undoped and doped ZnO materials were analyzed using various characterization techniques to evaluate their morphological and structural properties and photosensing capabilities. The surface morphology of the films was examined by scanning electron microscopy (SEM; Zeiss Evo LS 10). Energy-dispersive X-ray spectroscopy (EDX), coupled with SEM, was used to confirm both the films’ elemental composition and the successful incorporation of Al and In dopants into the ZnO lattice. The films’ thicknesses were quantified using the profilometer (AEP Technology NanoMap-500 LS 3D), with measurements taken from various sections of the samples. X-ray diffraction (XRD, Bruker D8 Advance) analyses were performed with Cu Kα radiation (λ = 1.5406 Å) to determine the crystal structure and phase composition of the films. Fourier transform infrared (FTIR) spectroscopy (Bruker Vertex 70) analyses were performed over the range of 400–4000 cm^–1^ to determine the functional groups and chemical-bond characteristics of the films.

Raman spectroscopy measurements were performed at room temperature using a Renishaw inVia confocal Raman microscope coupled to a Leica optical microscope with a 100× objective. A 532 nm green laser source with an output power of 30 mW was used for excitation, with an acquisition time of 1 s and 250 accumulations to improve the signal:noise ratio. Photoluminescence (PL) analyses were conducted using an Edinburgh Instruments FS5 spectrometer. An initial excitation wavelength scan identified the optimal excitation wavelength as 310 nm. Subsequent PL measurements were therefore recorded at an excitation wavelength of 310 nm with emission spectra collected from 350 to 600 nm in 1 nm steps.

Ti (50 nm) and Au (90 nm) metal contacts were used as current collectors on the surface of pure ZnO films and of ZnO films doped with Al and/or In. These contacts were produced by DC magnetron sputtering and thermal evaporation in a physical vapor deposition (PVD) system (NANOVAK NVTS-400). DC magnetron sputtering was used to deposit Ti, and thermal evaporation was used to deposit Au. A 99.99% (4N) pure sputtering target was used for Ti, and 99.9% (3N) pure pieces were used for Au. To achieve the desired Ti and Al patterns on the surface of doped and undoped ZnO films, a metal contact mask was placed on the films and the samples were then inserted into the PVD chamber. Ti coating conditions were achieved using a working pressure of 2 × 10^–6^ Torr and a production pressure of 4 × 10^–3^ Torr, with 10 sccm of 5N purity Ar gas supplied to the chamber. Metal contacts were deposited at room temperature with a 40 W DC power source at a rate of 0.5 Å/s. After the Ti deposition was completed, the power and Ar flow were shut off and the system was maintained under vacuum for a short period before the Au layer was deposited on the Ti. For the Au layer, the coating conditions were a production pressure of 1 × 10^–7^ Torr, a power of 90 W, and a deposition rate of 1.7 Å/s. Electrical measurements on the completed photosensors were performed using a Wonatech Zive SP1 potentiostat/galvanostat. *I–V* measurements were performed in the dark and under UV illumination at a scan rate of 250 mV/s over the range of −5 to 5 V. Sensor performance tests were conducted in potentiostatic mode with a period of 0.2 s, a stability of 10 mV/s, and a current resolution of 1 μA. Additionally, a UV light source with a wavelength of 400 nm and a light intensity of 75 μW/cm^2^ was used for measurements under illumination.

## 3 Results and Discussion

### 3.1 Morphological Analyses

The surface morphologies of the produced undoped and doped ZnO thin films were examined by SEM at 25,000× and 100,000× magnifications (Figure 2A–F). As shown in Figure 2A–F, significant changes in grain size, surface texture, and particle distribution were observed as a function of the Al and In content.

**Figure 2:**
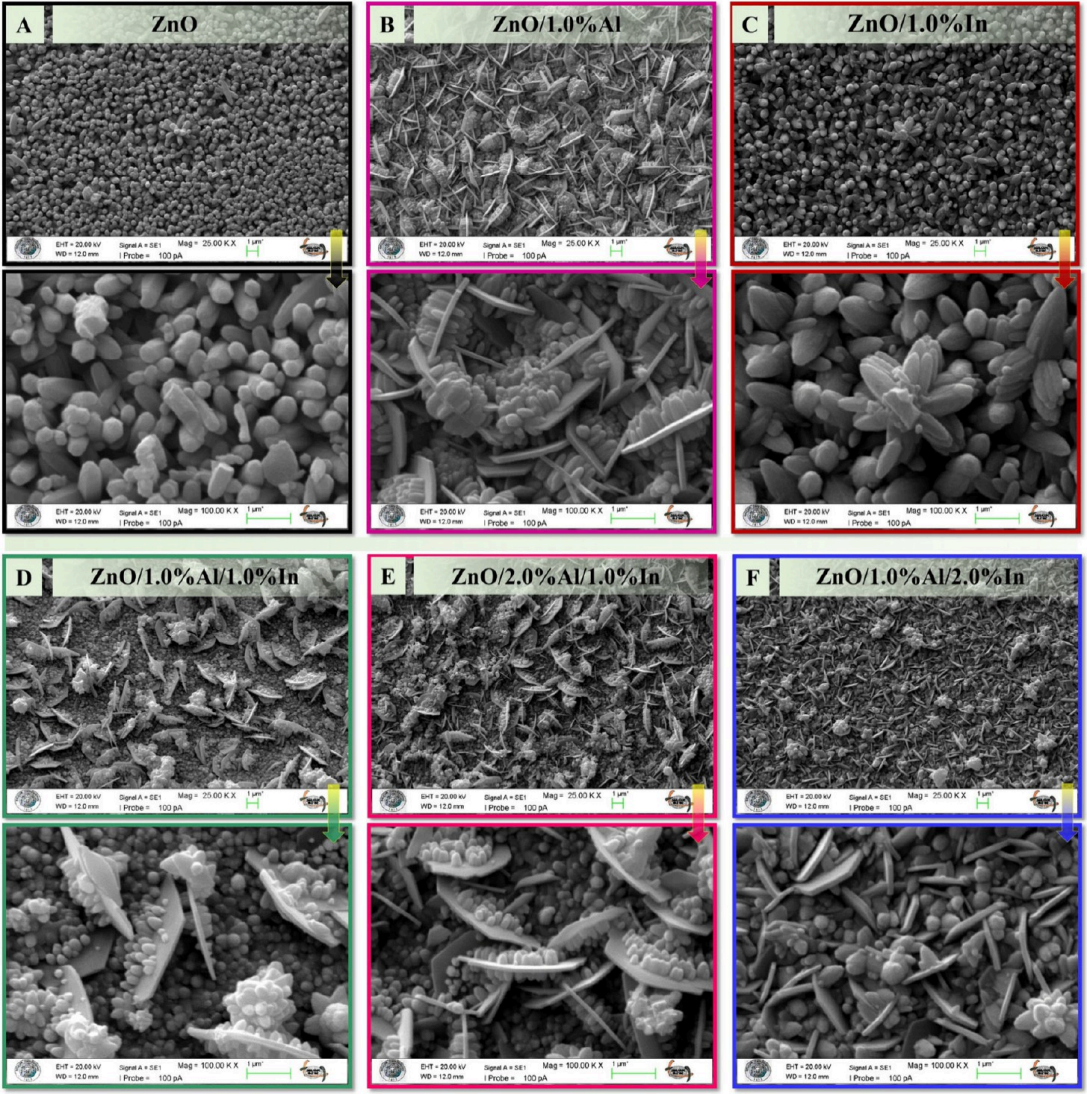
SEM images of undoped and doped ZnO thin films show changes in the surface morphology depending on Al and In doping. The images were taken at magnifications of 25,000× (top) and 100,000× (bottom).

The undoped ZnO film (Figure 2A) exhibited a homogeneous, dense, and compact grain morphology. The grains are hexagonal, rod-like structures with smooth surfaces and low porosity. With the addition of Al (Figure 2B), the surface morphology transformed into a layered, flake-like structure. It consists of flower-like, tightly clustered nanoparticles formed from thin hexagonal structures in doped ZnO (Figure 2C).

The observed morphological changes result from the influence of the doping elements on the nucleation and growth kinetics. In singly doped ZnO, substitution of Zn^2+^ (0.74 Å) with Al^3+^ (0.53 Å) or In^3+^ (0.80 Å) induces different types of stress in the ZnO lattice. Al^3+^ ions create compressive strain due to their smaller ionic radii and promote preferential growth along specific crystallographic directions, leading to the formation of flake-like structures. By contrast, In^3+^ ions create tensile stress because of a larger ionic radius mismatch, limit atomic mobility, and cause the formation of smaller, flower-like grains. Both of these contributions significantly transform the crystal growth mechanism and surface morphology of ZnO films by altering nucleation kinetics and surface energy.^24,26^

SEM images of dual-doped ZnO thin films (Figure 2D–F) show that increasing the doping ratio significantly alters the surface morphology due to the combined effects of Al^3+^ and In^3+^ ions on the ZnO lattice. A mixture of flake-like and hexagonal granular structures has been observed. The combined presence of Al and In induces complex stresses in the lattice, leading to a multifaceted surface morphology. At higher Al ratios, the surface became more compact and homogeneous. However, an increase in the In ratio has led to the formation of thicker flower-like structures. The combined contributions of Al and In have led to significant changes in the surface morphology by establishing a balance between nucleation and crystal growth mechanisms during the growth of ZnO films. The interaction between these two elements affects the stress, surface energy, and diffusion behavior in the ZnO lattice, thereby regulating the grain size, shape, and density.^27^

The average thickness of the hexagonal rod-like structures observed in the undoped ZnO film was approximately 400.1 nm, whereas this value decreased to 201.6 nm in the ZnO film doped with 1.0% Al and 1.0% In (Figure 3). This pronounced thinning resulted from Al^3+^ and In^3+^ ions settling into the ZnO lattice, thereby increasing the nucleation rate and limiting grain growth. In other words, particle refinement arises from modification of the nucleation and crystal growth kinetics of the dopant atoms during the SILAR process. The Al^3+^ ion (smaller radius) creates compressive stress, while In^3+^ (slightly larger) creates tensile stress. These lattice mismatches increase the nucleation density and limit long range crystal growth. As a result, the system transitions to a nucleation-dominant regime, and smaller particles form. Dual doping further enhances this effect by increasing the defect density and limiting atomic diffusion.

**Figure 3:**
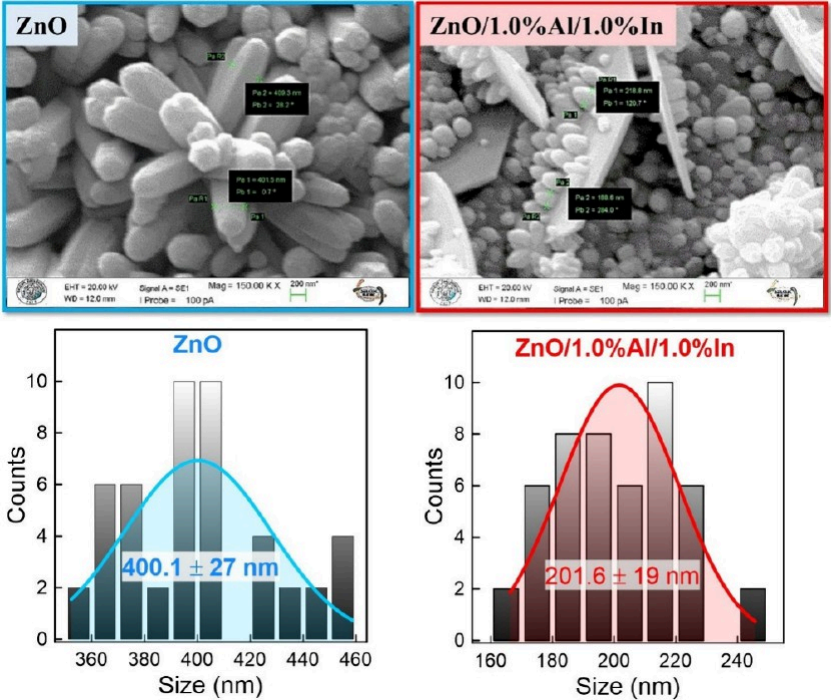
SEM images of pure ZnO and doped ZnO/1.0% Al/1.0% In films. One can see that the particle thickness decreased from 400.1 ± 27 to 201.6 ± 19 nm with doping.

The grain size distribution histogram, derived from SEM images, is shown in Figure 3 to quantitatively evaluate the surface morphology and microstructural uniformity of the undoped and doped ZnO thin films. This analysis enables us to evaluate the effect of doping on grain growth and morphology. It also enables us to correlate grain size with the functional properties of our fabricated samples, such as electrical conductivity and sensing efficacy. The reduction in particle thickness from 400.1 ± 27 to 201.6 ± 19 nm significantly increases the surface:volume ratio. For nanorod structures, reducing the diameter by approximately half increases the surface area per unit volume by approximately 2-fold. Since the photoresponse of ZnO is largely dependent on surface oxygen processes and defect-induced traps, increasing the surface area improves charge separation and extends the carrier lifetime. This leads to an increase in photoresponsivity.^28^ Furthermore, a significant increase in the number of grain boundaries occurs, and shrinking nanostructures can also lead to the formation of additional trap centers.

These morphological findings are also strongly supported by the XRD patterns. Combined XRD and SEM analyses indicate that the addition of Al and In produces a synergistic effect on the crystal structure and surface morphology of ZnO films, altering the orientation, stress, and grain structure. The peak shifts and intensity changes observed in the XRD patterns corroborate the differences in grain shape, size, and surface texture, as determined by SEM.

The EDX spectrum and elemental distribution (mapping) images of the ZnO/1.0% Al/1.0% In dual-doped sample in Figure 4 clearly show the presence and homogeneous distribution of Zn, O, Al, and In on the film surface. Strong peaks in the spectrum corresponding to Zn and O confirm the ZnO-based structure of the film, while lower-intensity yet distinct peaks corresponding to Al and In indicate that the dopant elements have been successfully incorporated into the ZnO matrix. The EDX mapping images in the lower section show a uniform distribution of elements across the surface with the O, Al, Zn, and In elements colored red, blue, green, and purple, respectively. This homogeneous distribution indicates that the diffusion of the additive elements throughout the film was successful and that no phase separation or surface clustering has occurred. Therefore, EDX analysis confirms that Al and In are simultaneously and effectively doped into the ZnO structure and that the double-doping process does not compromise the structural integrity.

**Figure 4:**
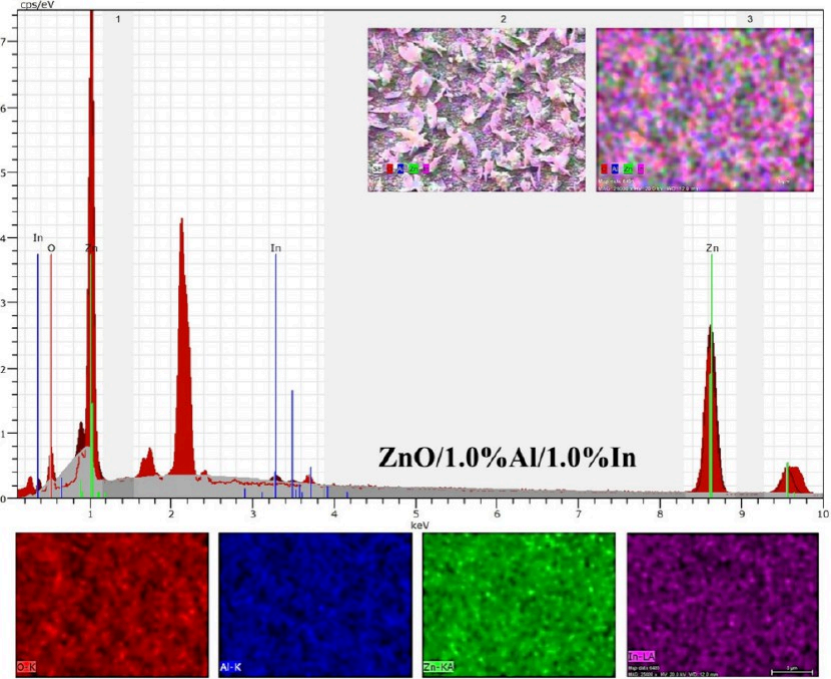
EDX spectrum and elemental distribution (mapping) images of the ZnO/1.0% Al/1.0% In dual-doped thin film show that Zn, O, Al, and In are homogeneously distributed across the film surface and that the dopants are successfully incorporated into the ZnO matrix.

### 3.2 X-ray Diffraction Analysis

The crystalline phase, particle size, and growth orientation of the samples were investigated by XRD. XRD analysis (Figure 5) revealed characteristic 2θ peaks for ZnO at 32.21°, 34.85°, 36.69°, 47.96°, 56.97°, and 63.23°. These peaks correspond to the (100), (002), (101), (102), (110), and (103) planes of the crystal lattice, respectively. The diffraction peaks obtained are consistent with the standard JCPDS Card 89-0510 data, confirming that the synthesized ZnO nanoparticles are in the hexagonal wurtzite crystal phase.^29^ The diffraction peaks observed in the undoped and doped samples are in good agreement with those in this reference file. Furthermore, no additional peaks belonging to Al_2_O_3_, In_2_O_3_, or other impurity phases were observed within the resolution limits of the device. This situation confirms that the dopant atoms have been successfully integrated into the ZnO lattice and that the main crystal structure has been preserved. The position and intensity of the (002) peak change with the doping level in all samples. Typically, a shift in peak position is observed when a dopant with an ionic radius different from that of the host cation (Zn^2+^) is substituted into lattice sites in ZnO. The Zn^2+^ ion (0.74 Å) has an ionic radius that is larger than that of Al (0.54 Å) but smaller than that of In (0.80 Å). The intensity of the main peak decreases when Al^3+^ is doped into the ZnO structure. This indicates that the Al^3+^ ion occupies interstitial sites in ZnO. The larger ionic radius of In relative to that of Zn enhances this strain effect. Furthermore, the ability of Al^3+^ to dissolve into the ZnO lattice is limited by the substantial size discrepancy between the Al^3+^ and Zn^2+^ ions. The substitution of dopant ions into the ZnO host lattice is confirmed by observed changes in dopant content and concentration. The primary crystalline structure of the ZnO samples was affected by doping with Al^3+^ and In^3+^, as made evident by SEM results.

**Figure 5:**
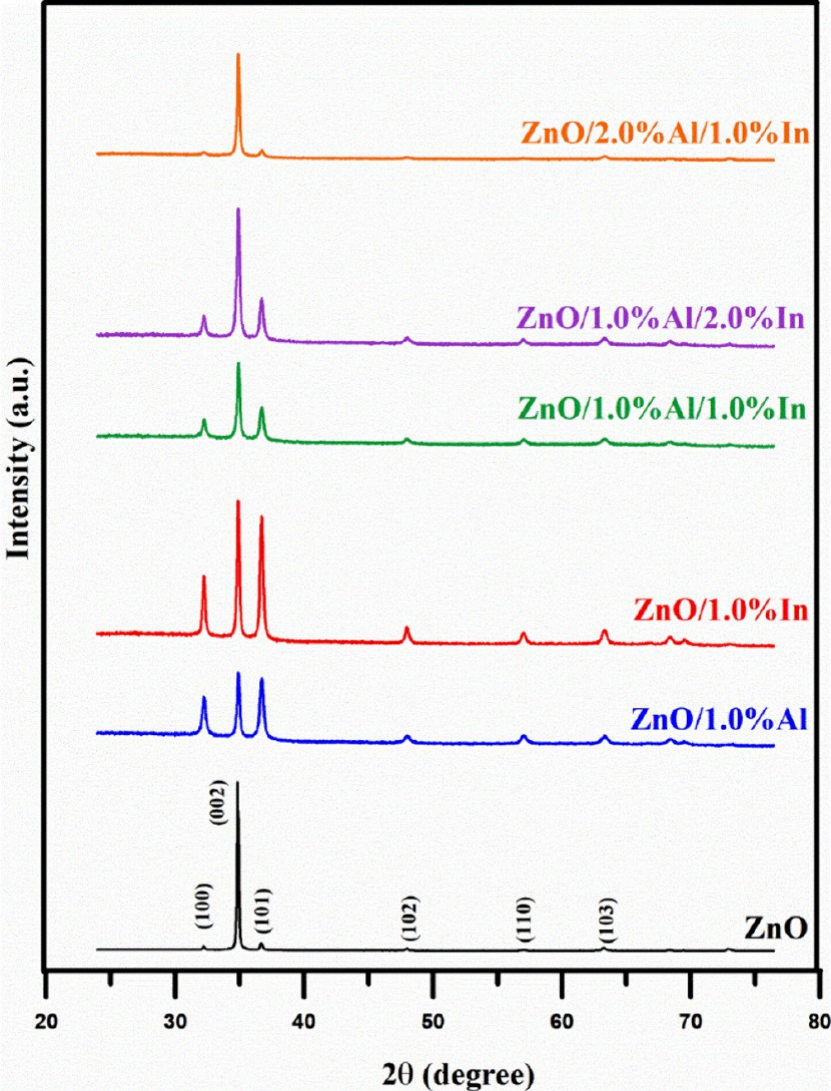
X-ray diffraction patterns of undoped and Al- and/or In-doped ZnO nanostructures. All of the samples show relative changes in the positions and intensities of the characteristic peaks as the doping type and concentration vary.

The crystallite size of undoped and doped samples can be calculated using Scherrer’s formula (eq 1)^30^

$$D=\frac{K\lambda }{\beta \;\cos\;\theta }$$ 1

where λ is the wavelength of the incident X-ray, β is the angular half-height width of the diffraction peaks, and θ is the Bragg diffraction angle. The calculated crystallite sizes were 54.96 nm for undoped ZnO, 38.51 nm for the Al-doped sample, 41.75 nm for the In-doped sample, and 38.66 nm for the dual-doped (Al and In) sample (Table 1). The change in the crystallite size of the ZnO film is evident and can be explained by Al and In ions being substituted into ZnO lattice sites. This results in a distortion of the lattice parameter, as previously reported.^31^ The SEM results also clearly demonstrate that doping alters the particle size and distribution.

**Table 1:** 2θ Values, fwhms, Crystallite Sizes, and *d*-Spacing Parameters of ZnO Samples, as Determined by XRD

sample	(*hkl*)	2θ (deg)	fwhm	crystallite size (nm)	*d*-spacing (nm)
ZnO	(002)	34.87	0.168	54.96	0.2570
ZnO/1.0% Al	(002)	34.90	0.240	38.51	0.2567
ZnO/1.0% In	(002)	34.88	0.222	41.75	0.2569
ZnO/1.0% Al/1.0% In	(002)	34.91	0.239	38.66	0.2567
ZnO/1.0% Al/2.0% In	(002)	34.90	0.260	35.59	0.2569
ZnO/2.0% Al/1.0% In	(002)	34.89	0.213	43.48	0.2568

High-crystallization quality and a well-ordered crystalline structure are indicated by the presence of low full width at half-maximum (fwhm) values and stable *d*-spacing parameters. *d*-spacing values that are consistent with standard crystallographic data confirm structural stability and minimal lattice distortion, whereas deviations may indicate internal stress or atomic substitution. As one can see, the change in this parameter is very small, suggesting that the quality of the resulting crystal structure is good. Additionally, the crystallite size is smaller in the doped samples compared with the undoped ZnO thin films. These results also agree well with the SEM results.

Changes in crystallite size are induced by internal lattice stress resulting from dual doping and variations in the dopant content and concentration. The host lattice is expected to be strained due to dopant-induced changes in the lattice parameters. The addition of Al and In ions to the ZnO crystallization process can alter the lattice parameters, unit cell volume, and crystallite size. These alterations can be attributed to the influence of both intra- and internucleating forces.^32^

### 3.3 FTIR Analyses

FTIR is a valuable technique that provides a detailed analysis of the vibrational modes of the synthesized samples. The various vibrational modes of Al- and/or In-doped ZnO samples were analyzed by using this method. The FTIR transmittance spectra of films are shown in Figure 6. This spectrum shows a few absorption bands in the fingerprint region (below 1000 cm^–1^) due to the ZnO characteristic stretching modes. The peaks appearing at ∼890, ∼765, and ∼675 cm^–1^ can be assigned to the characteristic stretching modes of ZnO and are consistent with the literature values determined using similar methods.^33,34^ Two peaks around 1530 and 1420 cm^–1^ can be assigned to the asymmetrical and symmetrical stretching modes, respectively, of the carboxylate groups (COO^–^) of the residual acetate moiety.^24,34,35^ Finally, the peak at 2350 cm^–1^ belongs to the absorbed atmospheric CO_2_.^34^

**Figure 6:**
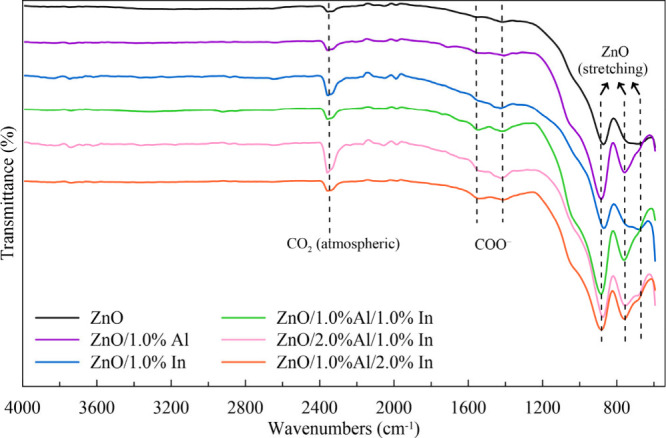
FTIR spectra of undoped and Al and/or In-doped ZnO thin films synthesized with varying Al and In doping levels.

The characteristic Zn–O vibrational bands observed in the fingerprint region confirm the formation of a ZnO lattice. Slight variations in these bands with Al and In doping indicate lattice distortion arising from the substitutional incorporation of dopant ions into the ZnO crystal structure (discussed in sections 3.4 and 3.5). Such structural distortions may lead to the formation of defect states, particularly oxygen vacancies, which play an important role in the visible photoluminescence emission of ZnO and act as trapping centers for photocarriers responsible for persistent photoconductivity.^9,36,37^

### 3.4 Raman Analyses

The Raman spectra of undoped ZnO and Al- and/or In-doped ZnO thin films shown in Figure 7a clearly reveal the characteristic vibrational modes of ZnO with a hexagonal wurtzite crystal structure. In the ZnO film, the dominant sharp peak observed at approximately 437–439 cm^–1^ corresponds to the E_2_ (high) mode, which is characteristic of the crystal symmetry of wurtzite ZnO. This mode arises from the vibration of oxygen atoms in the ZnO lattice and is widely regarded as an indicator of high crystalline quality. Table 2 summarizes the E_2_ (high) and E_1_ (LO) peak parameters extracted from the Raman data.

**Table 2:** E_2_ (high) and E_1_ (LO) Peak Parameters Calculated from the Shift in Raman Spectra and Degrees of Crystallization of Samples (*X*_c_)

	E_2_ (high)			E_1_ (LO)			
sample	position (cm^–1^)	fwhm (cm^–1^)	area (×10^6^)	position (cm^–1^)	fwhm (cm^–1^)	area (×10^5^)	*X*_c_
ZnO	438.25	8.58	2.857	581.44	13.01	3.518	89.04
ZnO/1.0% Al	438.25	11.69	1.426	579.59	13.68	10.88	56.74
ZnO/1.0% In	438.25	9.44	2.142	581.44	12.97	3.765	85.05
ZnO/1.0% Al/1.0% In	436.37	11.12	0.977	579.59	13.67	3.035	76.30
ZnO/1.0% Al/2.0% In	438.25	11.13	1.523	581.44	13.69	8.056	65.40
ZnO/2.0% Al/1.0% In	438.25	11.06	1.172	581.44	13.42	8.923	56.78

**Figure 7:**
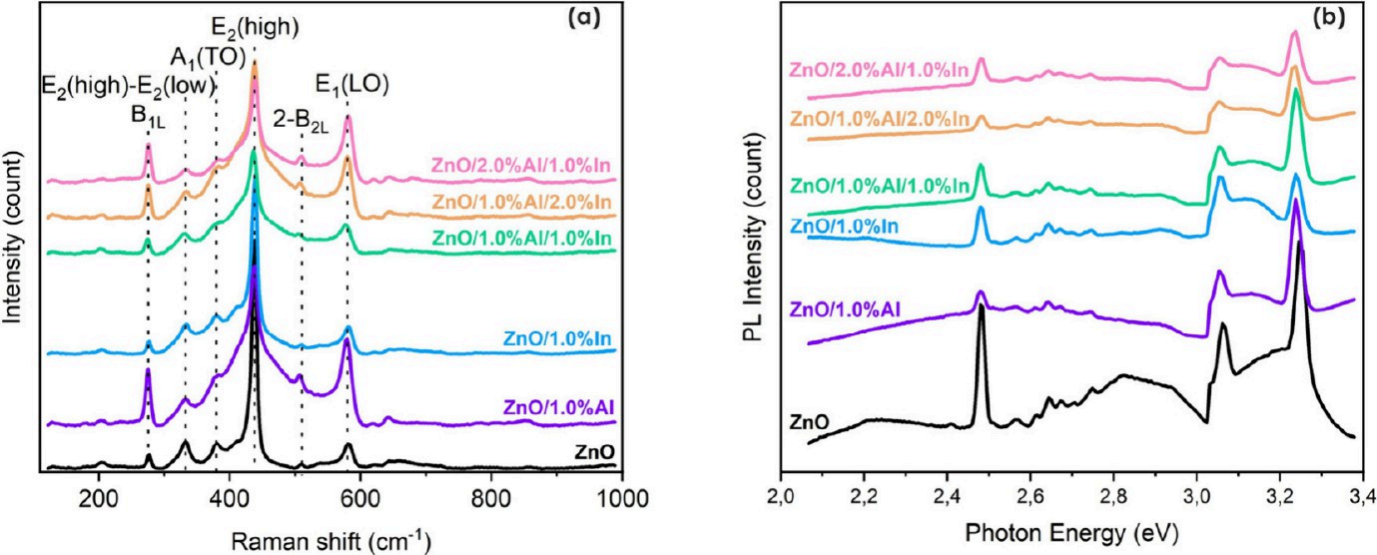
(a) Raman spectra of undoped, Al-doped, and/or In-doped ZnO thin films synthesized with varying In and Al doping levels. (b) PL spectra of undoped, Al-doped, and/or In-doped ZnO thin films synthesized with varying In and Al doping levels.

The E_2_ (high) peak is located at 438.25 cm^–1^ for undoped ZnO and remains near this value for most doped films, while a distinct red-shift to 436.37 cm^–1^ is observed for ZnO/1.0% Al/1.0% In. This shift is consistent with the dopant-induced lattice strain and local symmetry perturbation caused by the substitution of Zn^2+^ ions with trivalent dopants. The fwhm of the E_2_ (high) peak increases from 8.58 cm^–1^ for ZnO to approximately 11–12 cm^–1^ for Al-containing compositions, indicating increased microstrain and structural disorder.

Scattering related to the E_1_ (LO) mode is known to increase with defect-induced disorder (such as oxygen deficiency) and with an increasing free-carrier density.^38,39^ The weak band observed at 330–335 cm^–1^ is attributed to the second-order E_2_ (high)–E_2_ (low) mode, while the peaks at ∼380–385 and ∼410–415 cm^–1^ correspond to the A_1_ (TO) and E_1_ (TO) modes, respectively. The feature at ∼275–280 cm^–1^ is assigned to the B_1_ (low) mode, which is normally Raman inactive but may become activated due to lattice defects, strain, or dopant incorporation.^38–40^ The persistence of ZnO-specific Raman modes in all doped samples confirms that no secondary phases (e.g., Al_2_O_3_ or In_2_O_3_) are formed and that the wurtzite structure is preserved.

The crystallization fraction derived from Raman peak areas was determined using eq 2

2 $$X_{c}\;(\%)=\frac{A_{E_{2}\left(\mathrm{high}\right)}}{A_{E_{2}\left(\mathrm{high}\right)}+A_{E_{1}\left(\mathrm{LO}\right)}}$$

It is 89.04% for undoped ZnO and decreases significantly with Al incorporation.

This behavior indicates that Al doping enhances LO-related defect scattering more strongly than In-only doping. Among the Al-containing films, ZnO/1.0% Al/1.0% In retains a relatively high *X*_c_ value (76.30%) while exhibiting a reduced E_1_ (LO) area compared with those of other Al-rich samples. This suggests a more balanced defect structure, where excessive LO-related disorder is suppressed while structural integrity is partially maintained.

### 3.5 Photoluminescence Analyses

PL spectra recorded at room temperature under 310 nm excitation using a xenon lamp (without a polarizer) are presented in Figure 7b. The spectra clearly reveal both near-band-edge (NBE) and defect-related optical transitions in undoped and doped ZnO thin films.

The dominant high-energy emission band located at ∼3.24–3.25 eV (≈384–382 nm) is assigned to NBE excitonic recombination, consistent with previously reported UV emission of ZnO at room temperature.^41^ A slight red-shift of the NBE peak from 3.25 eV (ZnO) to 3.24 eV (doped films) is observed, which can be attributed to dopant-induced lattice strain and defect-carrier interactions rather than phase transformation.^42^ A secondary emission band at ∼3.05–3.06 eV (≈406–404 nm) corresponds to the violet deep-level emission (DLE) component and is commonly associated with shallow donor–deep acceptor transitions involving intrinsic point defects.^43^ The gradual reduction in the intensity of this band with an increase in dopant concentration indicates partial passivation or a redistribution of intrinsic defect states.

In addition, the spectral “valley” between the violet emission (∼3.05 eV) and the NBE peak (∼3.24 eV) exhibits a pronounced doping-dependent evolution. The intermediate-energy emission in the range of ∼3.10–3.20 eV becomes relatively more pronounced in the In-doped sample, whereas Al-containing and codoped compositions display a reduced midband contribution. Within a deep trap-dominant framework, this suppression of intermediate emission is not merely indicative of defect passivation but rather suggests an increased density of deep trap centers that efficiently capture photogenerated carriers before they can recombine radiatively through shallow defect states. In this case, a larger fraction of carriers is diverted toward nonradiative recombination channels, thereby reducing the radiative intensity in the intermediate region. This interpretation is consistent with the observed reduction of the green luminescence band (∼2.45 eV) and the redistribution of defect-related recombination pathways upon Al incorporation. Such behavior indicates that doping modifies not only the concentration of intrinsic defects but also their recombination dynamics, favoring deeper trap-assisted carrier capture over shallow radiative transitions.^44^

In the low-energy region, a broad emission band centered at ∼2.45 eV is clearly observed, particularly strong in undoped ZnO. This band corresponds to the well-known green luminescence of ZnO and is attributed to deep-level defect-related radiative recombination, commonly associated with oxygen vacancies (V_O_), zinc vacancies (V_Zn_), and related defect complexes.^45^ The relatively high intensity of this green emission in undoped ZnO indicates a high concentration of radiative deep-level defect centers. Upon Al or In incorporation, a pronounced reduction in green emission intensity is observed, indicating modification of the defect chemistry and a decrease in radiative deep-level recombination channels.

Group III dopants such as Al^3+^ and In^3+^ substitute for Zn^2+^ ions and act as donor impurities, increasing carrier concentration and modifying the balance between band-edge and defect-related recombination processes. An increasing dopant concentration leads to a reduction in visible defect-related emission and modifies the NBE:DLE intensity ratio, shifting recombination toward band-edge emission.^38,46^

The intermediate spectral region between ∼3.0 and ∼3.2 eV also changes with doping. The relative intensity of this region increases in In-doped ZnO, suggesting a higher density of radiative band-tail or shallow defect states. In contrast, Al-containing and codoped samples show reduced intermediate emission, indicating possible conversion of certain defect states into nonradiative recombination centers.

### 3.6 Optoelectronic Characterization and Memory Dynamics of ZnO-Based Devices

Photosensor devices were fabricated on ZnO-based thin films by creating nested contact patterns according to an interdigitated electrode (IDE) model. Figure 8a shows the standard current–voltage (*I–V*) characteristics of undoped, Al-doped, In-doped, and dual-doped (Al and In) ZnO films with Ti/Au metal contacts. The films were scanned in the dark under a UV light source (75 μW/cm^2^) with a peak wavelength of 400 nm and an applied voltage between −5 and 5 V. Figure 8b illustrates the device architecture of the IDE mask designed to connect the metal contacts to the doped and undoped ZnO films. The light exposure area created by this mask on the film surface is 29.72 mm^2^ (Figure 8b). Figure 8c shows the comparative *I–V* characteristics, measured under UV illumination, of undoped ZnO and a ZnO film doped with 1.0% Al and 1.0% In; the dual-doped film exhibits the highest photocurrent. In this context, the results of the analysis indicate a significant increase in the photocurrent of all films under UV illumination, suggesting a strong photoresponse of the films. Under UV illumination, the device doped with 1.0% Al and 1.0% In generated an approximately 11-fold higher photocurrent than the undoped ZnO device. This situation is desirable for detectability, as it separates the signal from noise and permits measurement of the response without requiring sensitive, costly amplifier circuits. As is well-known, excitation of a material by light with energy close to the band gap promotes electrons from the valence band to the conduction band, resulting in the formation of electron–hole pairs.^37^ These electrons directly contribute to the photocurrent. The Ti/Au electrode ensured the rapid return of these electrons to the electrode surface. The *I–V* characteristics are approximately linear and symmetrical in the forward and reverse bias regions, indicating that ohmic conduction is the dominant mechanism at the interface between doped and undoped ZnO films and Ti/Au electrodes.^47,48^ It has been reported that Schottky behavior is dominant when Au is applied alone to ZnO.^49^ In this study, gold (Au) was deposited onto the Ti layer to prevent the oxidation of Ti. Thus, using the Ti interlayer makes the ohmic behavior dominant. The dark current value was higher than that of the undoped ZnO film (4.35 × 10^–6^ A) for all films except the dual-doped ZnO film (1.0% Al and 2.0% In) (3.81 × 10^–6^ A). Similarly, in the UV medium, higher photocurrent values were recorded for all films than for the undoped ZnO film (1.92 × 10^–5^ A), except for the 1% Al-doped ZnO film (1.47 × 10^–5^ A). Among all films under UV illumination, the ZnO film doped with 1.0% Al and In exhibited the highest photocurrent, 2.11 × 10^–4^ A. The next highest values were observed in 1.0% In-doped ZnO (1.30 × 10^–4^ A), 2.0% Al and 1.0% In dual-doped ZnO (3.11 × 10^–5^ A), and 2.0% Al and 1.0% In dual-doped ZnO (2.02 × 10^–5^ A) films, in that order. Under illumination, the current–voltage characteristic becomes asymmetric, and the difference between the dark current and the photocurrent increases as the voltage approaches 5 V. This is because the current increase process, which begins with the activation of light at −5 V, causes this difference as the duration increases toward 5 V. However, except for the dual-doped ZnO film (1.0% Al and 2.0% In) measured in the dark and the 1.0% Al-doped ZnO film measured under UV, all other doped ZnO films exhibited current values higher than those of the undoped ZnO film. These two films exhibit lower current values than the undoped ZnO film, which may be due to a decrease in electron concentration, a reduction in the effective carrier density, an increase in structural disorder, and an increase in the number of defects.^50^

**Figure 8:**
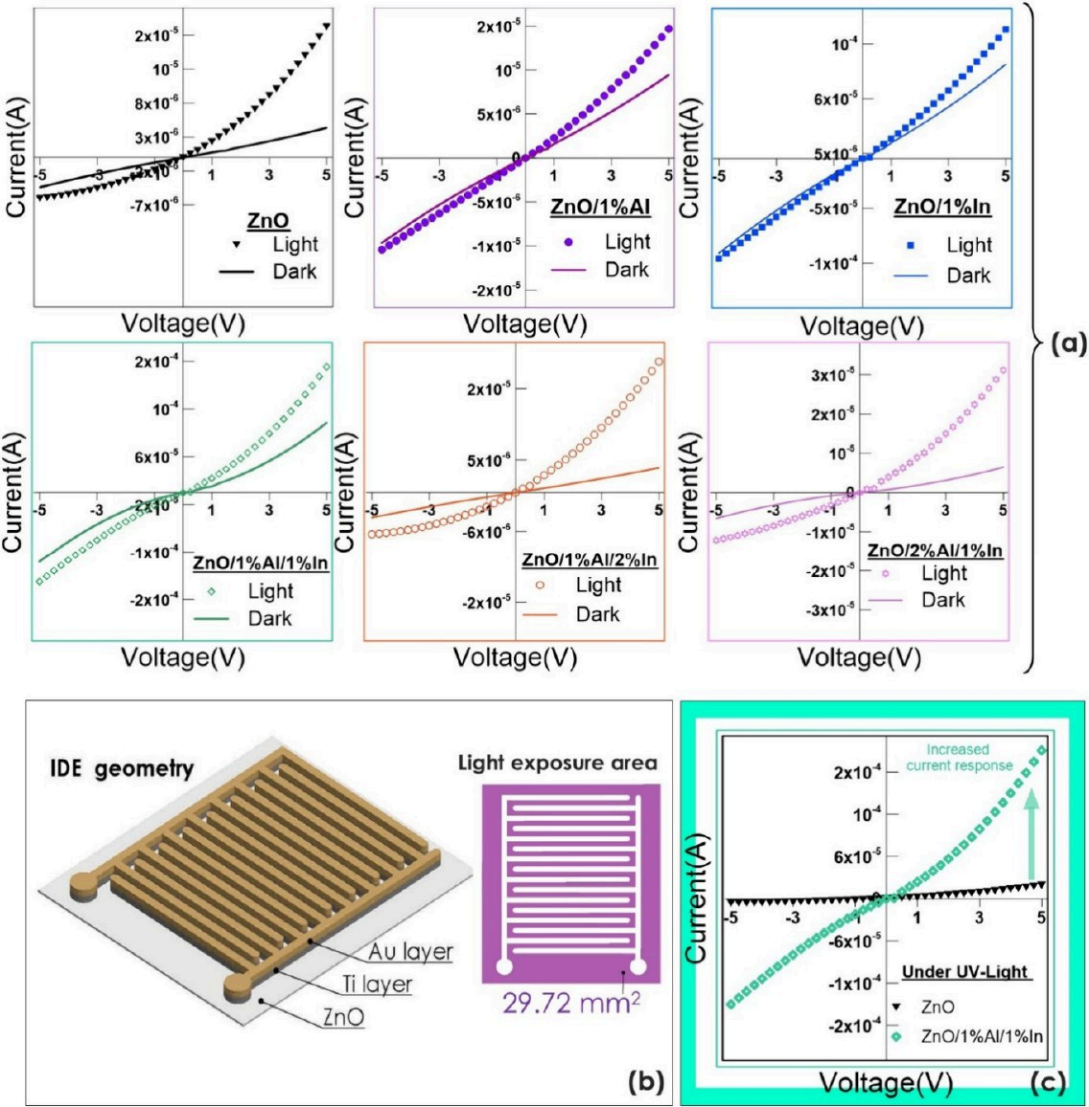
(a) Current–voltage characteristics of ZnO-based photosensors in the range of −5 and 5 V in the dark and under UV light. (b) Schematic geometry of the Ti layer deposited by sputtering on thin films and the Au layer formed by thermal evaporation. (c) Comparative current–voltage characteristics of undoped and 1.0% Al- and In-doped samples under light.

On the other hand, the superior electrical properties of the 1.0% Al and In dual-doped ZnO film can, in both cases, be attributed to its high surface roughness, suitable film thickness, differences in crystal structure, defect density, and changes in trap mechanisms.^51–53^ Indeed, the variation in XRD peak intensities and the differences in lattice parameters suggest that Al^3+^ and In^3+^ ions were incorporated into the lattice, introducing additional electrons into the system and contributing to the increased current. A similar mechanism has been previously reported for Sm doping of ZnO.^52^ In addition, the high current can lead to the absorption of photons scattered from UV light by oxygen molecules, releasing oxygen and producing free electrons, thereby increasing the photocurrent.^47^ The *I–V* curves suggest that the optimal doping ratio is 1.0% Al to 1.0% In. The doping process alters both the trapping mechanism and the free-carrier density. Further analyses were performed to examine the mechanisms of photocurrent generation, observe photonics-based memory effects, determine photosensor properties and device sensitivity, and assess trap effects.

The effect of single or binary doping on the photoconductive behavior of ZnO thin film materials in air is shown in Figure 9a. During the experiment, all films were exposed to UV light for 60 s. At the end of this period, the UV light source was turned off, and the films were kept in the dark for 600 s to examine the time-dependent change in current. Upon initial exposure to UV light, the photosensors exhibited an increase in photocurrent. However, photocurrent saturation was not reached due to the simultaneous desorption of oxygen molecules, resulting in the formation and collection of electron–hole pairs.^47^ In addition, when exposure to the UV light source ends, the current decreases gradually and does not return to a stable state. The primary reason for the inability of the photosensor to return to a stable state is the slow rate of adsorption of oxygen molecules, which indicates a reduced charge-transfer rate.^47,54^ Although all photosensors exhibit a slow degradation rate after UV light exposure for 60 s, their inability to return to a stable state even after prolonged periods supports the PPC property. According to the results, the lowest photocurrent was observed in 2.0% Al and 1.0% In dual-doped ZnO films, while the highest photocurrent was observed in 1.0% Al and 1.0% In dual-doped ZnO films. Other Al, In, and dual-doped (Al and In) films exhibited a higher photocurrent than the undoped device. Figure 9b shows decay times τ_1_ and τ_2_, obtained from the decay portion of the time-dependent photocurrent shown in Figure 8a, following the removal of UV light. These decay transition times were calculated using the double-exponential function described below (8,9):

3 $$I_{\mathrm{dec}}\left(t\right)=I_{0}+A_{1}\left(e^{-t/\tau_{1}}\right)+A_{2}\left(e^{-t/\tau_{2}}\right)$$

where *I*_0_ is the dark current, *A*_1_ and *A*_2_ are constants, and τ_1_ and τ_2_ are time constants. In Figure 9a, the experimental data are shown as straight lines while the fits of the double-exponential equation (eq 3) are shown as symbols matching the color of each sample. The graph shows that the fitted curves agree well with the experimental results. The characteristic decay times obtained by fitting the data to exponential functions are shown in the bar graph in Figure 9b.

**Figure 9:**
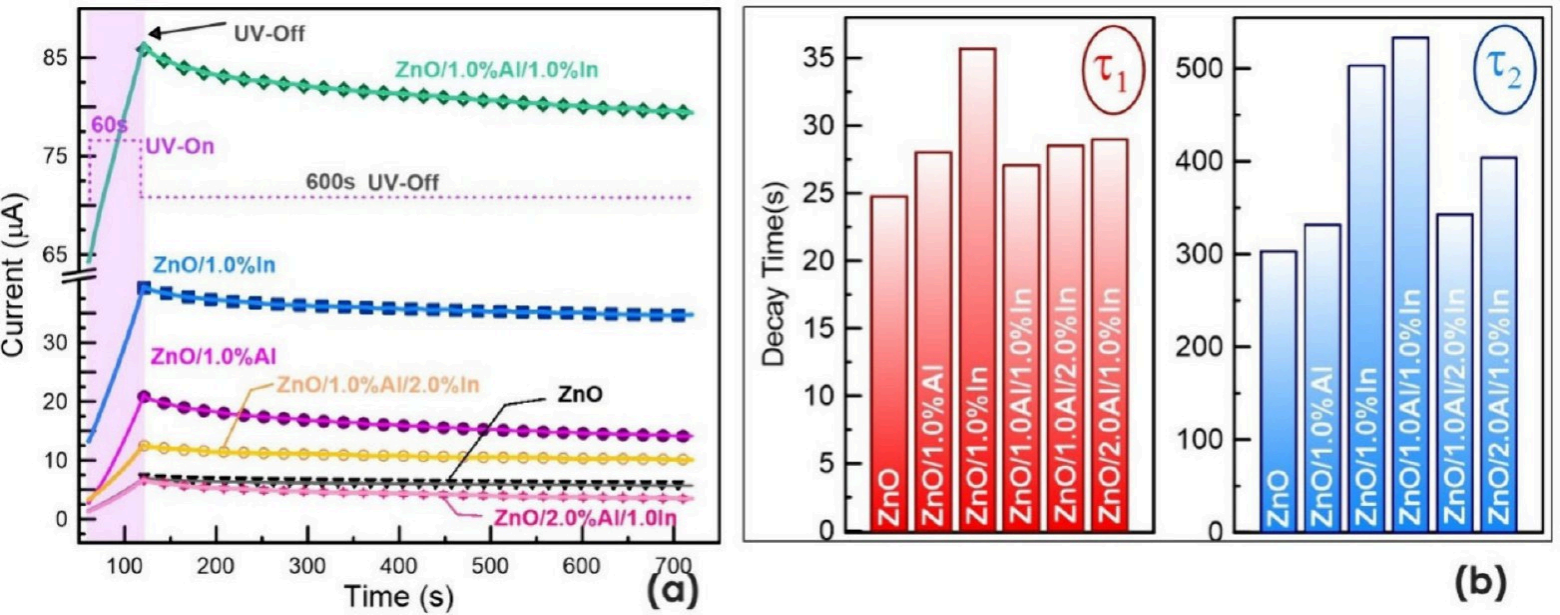
(a) Dark current decay characteristics and dual-exponential fits of the decay portion of ZnO-based photosensors after UV exposure for 60 s for 5 V bias at 600 s. (b) Bar plots of characteristic decay times (fast decay part τ_1_ and slow decay part τ_2_).

A two-stage current decay is observed in the devices after the UV light is switched off. First, the fast exponential component, represented by τ_1_, arises primarily from the rapid coupling of free electrons in the conduction band with untrapped holes. Additionally, oxygen molecules on the surface rapidly capture electrons, resulting in a decrease in the current. In the bar graph in Figure 9b, this portion exhibits similar behavior across all samples. Only the ZnO/1.0% In sample differs slightly. This suggests surface irregularities that increase short-term carrier retention due to doping.^50^ Overall, one can conclude that the responses of all samples to the initial shock caused by the light shutdown are similar, doping does not have a significant impact on the material’s main characteristics, the contact structures do not differ, and no effects disrupt the crystal structure. The portion represented by τ_2_ is the slow component of the exponential decay of the current. This portion represents the device’s long-term retention of photogenerated carriers. The device’s dominant current memory mechanism can be visualized from this portion. This allows the UV signal to evolve from a simple trigger into a state that contains optical information. Thus, one can find applications in next-generation optical computing systems. During this slow decay phase, the devices become more differentiated, as illustrated in Figure 9b, where the highest τ_2_ value, 533 s, is achieved for the ZnO device doped with 1.0% Al and In. This indicates that this doping ratio is optimal, providing an appropriate balance between trap-assisted retention and carrier production. This occurs because deep trap regions introduced by doping within the material’s crystal structure can capture photogenerated holes, preventing their migration to the surface. Thus, it is likely that electrons in the conduction band take a long time to recombine with trapped holes, resulting in a permanent conductivity effect and enabling the device to retain a memory of the current. The photocurrent performance exhibited by the ZnO/1.0% Al/1.0% In device is comparable to that of current 2D-based optoelectronic synaptic devices (OSDs) in the literature. For example, in the reported Bi_2_O_3_ content 2D-based study, it was observed that the current could maintain 62% of the initial level after 500 s following a 100 s optical stimulation, and this was described as nonvolatile memory behavior.^55^ Notably, the ZnO/1.0% Al/1.0% In system developed in this study maintained a level above ∼92% of the peak current for 600 s after stimulation was stopped, with a shorter exposure time of 60 s. This also shows that the short exposure time, with a light intensity of ∼75 μW/cm^2^, allows the device to operate with a low power consumption of ∼4.5 mJ/cm^2^ and be excitable (writable) with this low optical dose signal. In modern optoelectronic devices, minimizing energy consumption with short exposure times is a desirable feature.^56^

In the SEM images, the rod-like nanostructures in ZnO were significantly altered by 1.0% In and 1.0% Al doping. This structural change, with an increased number of grain boundaries and increased surface roughness, may have created additional trap centers. Thus, these structural obstacles may have prolonged the return of the carriers, which is supported by SEM analysis. Pujar et al.,^37^ in their studies of Sn-doped ZnO produced by SILAR, attribute the different decay times observed after doping to changes in surface morphology. Additionally, the characteristic decay times for all samples and the results reported in similar studies are presented in Table 3. Factors that can affect the decay time, such as exposure time, bias voltage, and fabrication techniques, are also included here. The results can be considered within a meaningful range. Decay times higher than many reported in the literature were obtained, and 1.0% Al and In dual doping successfully enhanced the memory effect of the ZnO-based device.

**Table 3:** Characteristic Decay Times of ZnO-Based Photosensor Devices and Comparative Values with Those in the Literature

sample	technique	bias (V)	UV exposure time (s)	τ_1_, fast decay time (s)	τ_2_, slow decay time (s)	ref
ZnO	SILAR	5	60	24.74	302.89	this work
ZnO/1.0% Al	SILAR	5	60	28.01	331.40	this work
ZnO/1.0% In	SILAR	5	60	35.68	503.13	this work
ZnO/1.0% Al/1.0% In	SILAR	5	60	27.07	533.31	this work
ZnO/1.0% Al/2.0% In	SILAR	5	60	28.50	342.64	this work
ZnO/2.0% Al/1.0% In	SILAR	5	60	28.98	403.83	this work
ZnO	SILAR	5	30	4.19	–	^51^
ZnO/10% Na	spray pyrolysis	5	60	2.30	14.3	^57^
ZnO/Ga	chemical bath	1	89	-	106	^58^
ZnO	sputter	1	1000	110.05	1353.11	^59^
ZnO/1.5% Al/1.5% Si	sol–gel	8	400	23.79	187.49	^60^

To visually represent the current memory of ZnO-based devices, a schematic representation was used in which the letter M (“memory”) was simulated as the region where light passed through the contact pattern. In Figure 10, the photocurrent obtained after UV illumination for 60 s, when UV light fell between the IDE contacts through a mask similar to those used in real experiments, was taken as 100%. A photograph of the manufactured ZnO device is shown. The simulation illustrates the decay of photocurrent over time from its 100% value on the color scale after the UV light is turned off. This visualization was generated for the value of τ_2_ of the permanent current memory portion. For the decay simulation, the current memory was related to color brightness, and the rate of decrease over time was calculated using the following equation:

4 $$\mathrm{brightness (\%)}=100\left(1-e^{-t/\tau_{2}}\right)$$

**Figure 10:**
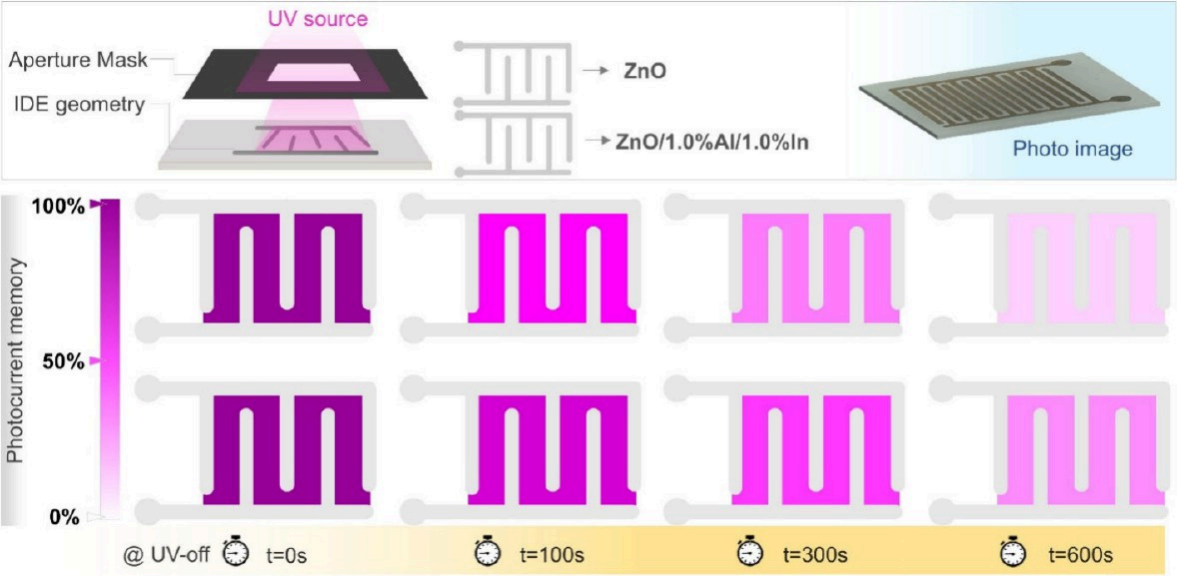
Diagram showing a schematic diagram of the UV application experiment on the devices, a photographic image of the ZnO photosensor, and a schematic representation simulating the permanent current memory of an undoped sample and a 1.0% Al and In dual-doped sample after UV exposure for 60 s followed by darkness for 600 s (it was assumed that the photocurrent memory started at 100% charge at the UV-off moment for both samples).

Equation 4 represents the increase in memory visibility but not the current itself. The slow decay values were calculated from the experimental results. A comparative visualization was used to compare a ZnO sample doped with 1.0% Al and In, which exhibited the longest τ_2_ decay time of 533 s, with an undoped ZnO sample, which had a value of 303 s. Within the first 300 s, a large portion of the slow PPC in the ZnO sample had decayed and most of its current memory had been depleted. In the ZnO/1.0% Al/1.0% In sample, however, the majority of the PPC remained and the current memory remained significant. After 600 s, the difference in current memory between the two samples was clearly evident from the color brightness, and the ZnO sample had reached its memory capacity.

This visualization effectively represents the PPC behavior and clearly illustrates the differences between the samples. It shows that the ZnO/1.0% Al/1.0% In sample exhibits a significantly prolonged PPC response relative to that of ZnO. It provides visual evidence that doping can extend the current memory of ZnO-based photonic memory devices. All of the other doped samples have higher τ_2_ values than ZnO. Specifically, the highest τ_2_ value of ZnO/1.0% Al/1.0% In indicates an abundance of deep traps, enhanced hole trapping, and an increased number of grain boundaries; SEM images support this interpretation. These mechanisms support the idea that electrons remain in the conduction band for an extended period, leading to current memory and slowing the return of electrons to their original states.

To determine the behavior of photonic memory devices over successive cycles, 12 on–off cycles were performed, each consisting of 60 s of UV on and 360 s of UV off. The results of these tests are listed in Figure 11. The photosensors’ photocurrent response exhibits a staircase-like increase over time. The photocurrent induced by light does not return to its baseline during the waiting period due to the memory effect. In these graphs, the gray traces on the right axis represent the semiderivative current $\left(\frac{d^{0.5}I\left(t\right)}{dt^{0.5}}\right)$ values. Semiderivative analysis enables us to visualize, mathematically, not only the speed of the current but also its variation arising from diffusion and trapping.

**Figure 11:**
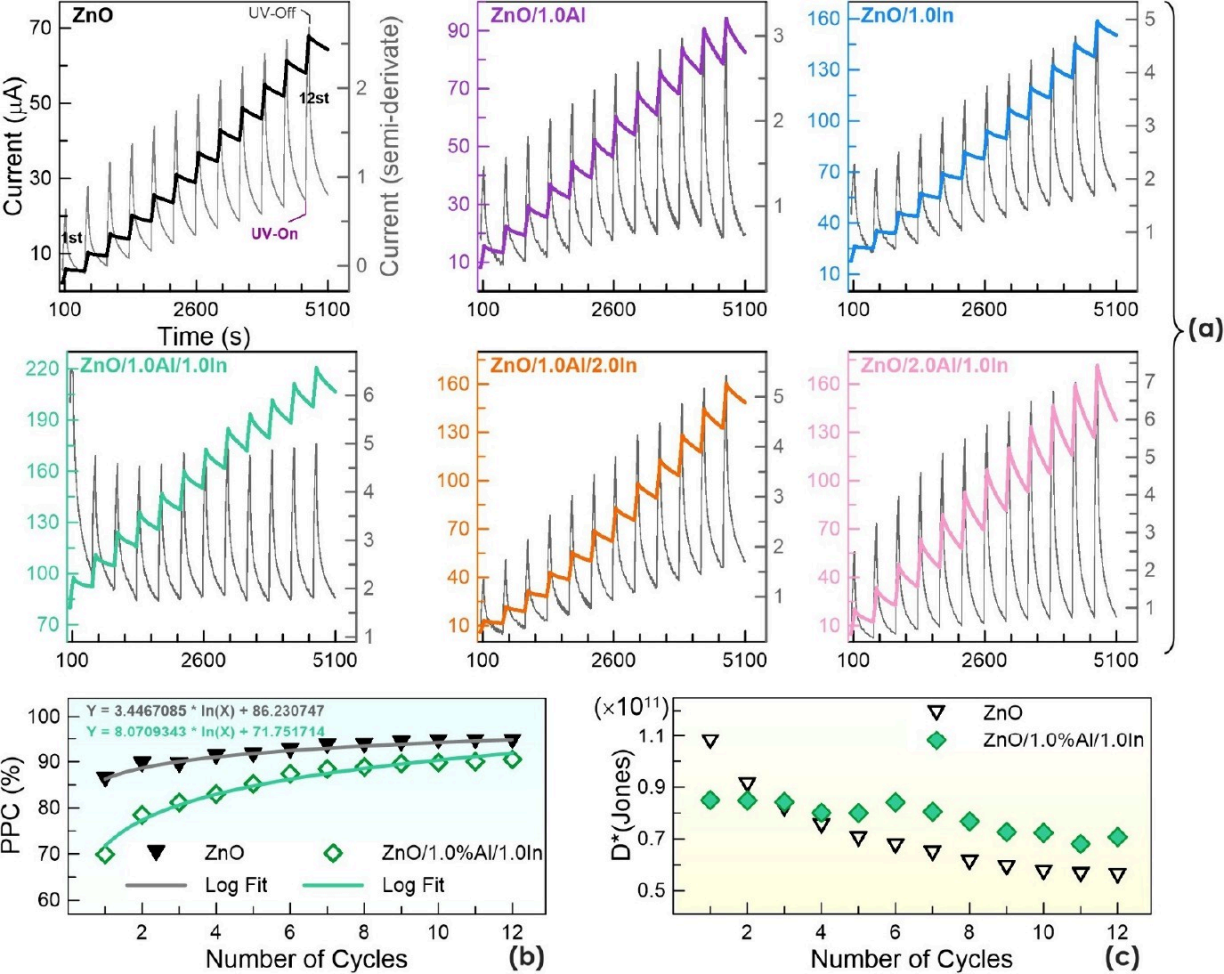
(a) Time-dependent current and semiderivative current variations of ZnO-based photosensor devices over 12 cycles with a 5 V bias voltage, 60 s of UV on, and 360 s of UV off. (b) Comparative graph and logarithmic fits of cycle-dependent PPC (%) values of undoped and 1% Al and In dual-doped samples. (c) Comparative cycle-dependent detectivity changes of the same device.

The semiderivative peak amplitude in ZnO varies between approximately 0 and 3 μA/s (with an amplitude below 1 μA/s in the initial cycles, increasing above 1 μA/s as the number of cycles increases). In contrast, the minima shift upward and the amplitude increases with the number of cycles. ZnO produces an unstable signal as the number of cycles increases, suggesting that signal detection may deteriorate over time due to residual charges and noise. In these tests, the 1.0% Al and In dual-doped ZnO sample stands out most, with stronger amplitudes of the semiderivative peaks and approximately constant maximum and minimum values as the cycles progress. Furthermore, the amplitudes of these peaks are more than twice those of ZnO and exceed 3 μA/s in almost all cycles. This indicates that the traps of the 1.0% Al and In dual-doped ZnO photosensor tend to acquire approximately the same amount of charge in each cycle and release it with similar stability. Based on its amplitude, one can conclude that it has a higher load capacity than ZnO. It can be assumed that it exhibits signal stability throughout the cycles without showing signs of fatigue or noise-induced chaotic behavior. The derivative amplitude of other doped samples, for example, the ZnO/2.0% Al/1.0% In sample, increased to a higher value after 12 cycles; however, the most stable cycle was still observed with the ZnO/1.0% Al/1.0% In sample.

Figure 10b shows the PPC% change of ZnO and 1.0% Al and In dual-doped devices over 12 cycles as a function of cycle number. PPC% values were calculated by using eq 5.

5 $$\mathrm{PPC\%}=\frac{I_{\mathrm{dec}}^{n}-I_{0}^{n}}{I_{\max}^{n}-I_{0}^{n}}\times 100$$

where *n* is the cycle number, *I*_0_^*n*^ is the latest current value before the light is switched on in each cycle, *I*_max_^*n*^ is the maximum photocurrent achieved upon UV exposure for 60 s, and *I*_dec_^*n*^ is the decay current at the end of 360 s after the UV is switched off. The initial dark current value was deliberately excluded to eliminate the influence of the previous cycle on measured current *I*_0_^*n*^.

PPC% values increase logarithmically and are well described by eq 6 (*R* ^2^ > 0.97).

6 PPC%(n)=Alog(n)+B

where coefficient *A* represents the memory effect, which depends on cycle log­(*n*), and constant *B* represents the initial PPC%. The 1.0% Al and In dual-doped device exhibits memory gain at a rate of change more than twice that of pure ZnO and catches up to pure ZnO, which initially exhibits a high PPC% value, after 12 cycles. The logarithmic behavior of PPC% supports the idea that pure ZnO reaches saturation quickly. At the same time, the sample doped with both 1.0% Al and In exhibits a high capacity, enabling longer-term charge accumulation supported by deep traps. Similarly, Bayan and Mohanta^16^ reported an increase in the loop-dependent PPC ratio. The increased memory effect was found to be also supported by using an excitation (light) source below (∼400 nm ≈ 3.10 eV) the fundamental band gap of ZnO (∼382 nm ≈ 3.25 eV). This is because the theoretical optical excitation threshold required to ionize the neutral oxygen vacancies in ZnO and switch it to metastable conductivity mode is 2.83 eV.^61^ The 3.10 eV energy of the UV source is sufficient to trigger structural relaxation and the resulting PPC effect, exceeding the conversion threshold (2.83 eV). Furthermore, it has been reported that excitation below the band gap provides a more homogeneous photon distribution across the film volume, thus being more effective in activating volumetric defects.^62^ Therefore, the UV source may have enabled the excitation of oxygen vacancies not only on the surface but also in the depths of the material, creating a more stable and high-capacity data storage potential. Furthermore, Figure 11c supports the finding that the specific detectivity values of ZnO decrease with an increasing cycle number, indicating a decrease in its detectability. In contrast, the specific detectivity of the 1.0% Al and 1.0% In dual-doped sample is not significantly affected by cycling. These results indicate the optimal doping ratio for ZnO doped with both 1.0% Al and 1.0% In and support its potential use in photonic memory applications, such as multilevel data storage and optical programming. For example, a previously reported ZnO-based study^63^ highlighted that the behavior under successive light pulses is crucial for implementing read/write-enhanced optical neural networks.

Figure 12 shows a schematic representation of the maximum photocurrent measured during each UV on–off cycle (60 s of light and 360 s of dark) in ZnO and ZnO/1.0% Al/1.0% In samples. In this diagram, photocurrent values are normalized to the maximum photocurrent of the ZnO/1.0% Al/1.0% In sample measured after 12 cycles and are represented on a color scale.

**Figure 12:**
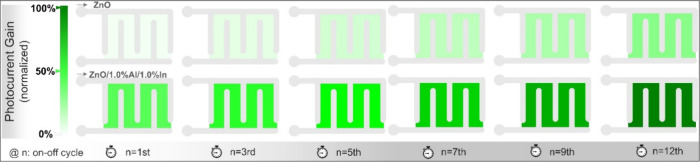
Comparative color schematic representation of photocurrent gains in a 12-cycle on/off test (maximum photocurrent values are normalized to the current reached by the ZnO/1.0% Al/1.0% In sample, which has the highest value, after 12 cycles).

The green color darkens as the current increases, beginning in the first cycle. The color change indicates that the photocurrent gain of the sample with a 1.0% Al:1.0% In doping ratio increases significantly from the first cycle onward. In contrast, the color scale clearly shows that the undoped ZnO sample, even after 12 cycles, exhibited a photocurrent lower than that measured in the 1.0% Al/1.0% In sample’s first cycle. Thus, both long-term decay tests and successive on–off tests indicate that a 1.0% Al and In doping rate is optimal for the Al- and In-doped set. The ZnO/1.0% Al/1.0% In device exhibits an enhanced ability to acquire current signals over successive cycles and retain this information during the prolonged decay period following UV exposure. In particular, the stepwise photocurrent response in each cycle and its high persistence in the ZnO/1.0% Al/1.0% In device indicate that this device has the potential to function as a nonvolatile photonic memory unit with sufficient data storage capacity. This potential can only be defined by following the architectural principles of integrated sensing memory units.^64^

To investigate the voltage-dependent changes of the sensors, measurements were taken on undoped and 1.0% Al and In dual-doped ZnO photosensors at bias voltages of 1 and 5 V. To examine the effects of the duration of light exposure, these photosensors were kept in the dark for 96 h before being measured. Figure 13a shows a sample measurement graph of the photosensors under a 1 V bias during UV light exposure for 5, 10, and 30 s. The semiderivative behavior was examined to characterize the photosensors’ light response, as in a monitor. In the same graph, the left vertical axis shows the current and the right vertical axis shows the semiderivative of the current. The current exhibited ladder-like behavior and retained residual components of the previous signal. To eliminate the effects of residual current of earlier cycles and isolate the impact of exposure time, the current was taken as the initial current during the first 5 s of supply; in subsequent cycles, it was taken as the minimum current value at the end of a 40 s light-off period, *I*_0_. The current value is the maximum current reached at the end of each exposure period, *I*_light_.

**Figure 13:**
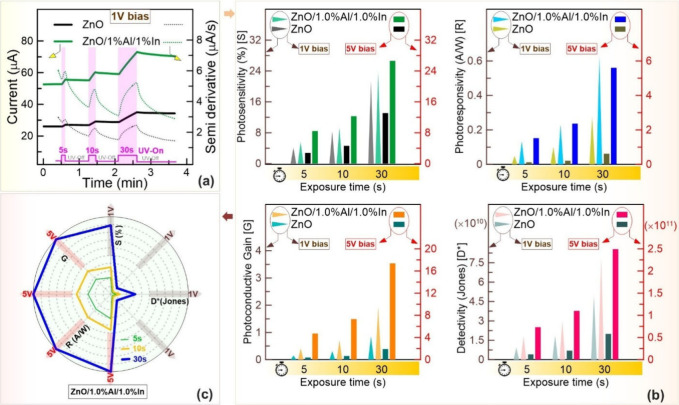
(a) Comparative current–time and semiderivative–time graphs of ZnO and ZnO/1.0% Al/1.0% In devices as a function of exposure time under a 1 V bias. (b) Comparative instrument performance indicators with biases of 1–5 V. (c) Radar graph reflecting the performance evaluation of the ZnO/1.0% Al/1.0% In sample, including exposure time at biases of 1 and 5 V (parameters normalized to the 5 V bias value).

Additionally, parameters in photosensors, such as photosensitivity, photoresponsivity, detectivity, and photoconductive gain, are essential criteria for evaluating device performance. Photosensitivity quantifies the sensitivity of photosensors and is calculated as follows (eq 7):

7 $$\mathrm{photosensitivity (}S)=\frac{I_{\mathrm{light}}-I_{0}}{I_{0}}\times 100$$

where *I*_light_ is the photocurrent and *I*_0_ is the dark current. Photoresponsivity, defined as the photocurrent produced per unit area of incident power, is calculated using the following formula (eq 8):

8 $$\mathrm{photoresponsivity (}R)=\frac{I_{\mathrm{light}}-I_{0}}{PA}$$

where *P* represents the light power (watts per square centimeter) and *A* represents the active area (square centimeters). Specific detectivity quantifies the ability to detect weak optical signals and is calculated as follows (eq 9):

9 $$\mathrm{detectivity}\;\left(D^{*}\right)=\frac{R}{\sqrt{2eJ_{0}}}$$

where *e* is the electron charge (1.6 × 10^–19^ C) and *J*_0_ is the dark current density (amperes per square centimeter). Photoconductive gain represents the number of effective photogenerated carriers produced per absorbed incident photon and is calculated by using the following formula (eq 10):

10 $$\mathrm{photoconductive gain}\;\left(G\right)=R\frac{hc}{\eta el}$$

where *h* is Planck’s constant (6.626 × 10^–34^ J s), *c* is the speed of light (3 × 10^8^ m/s), η is the quantum efficiency (1 is used for the sake of simplicity),^65^ and *l* is the wavelength of UV light (∼400 nm). The calculated parameters are shown for comparison in the bar-column plot in Figure 13b.

The corresponding values are given in Table 4. Photosensitivity, photoresponsivity, sensitivity, and photoconductive gain all increased with longer UV light exposure times (5, 10, and 30 s) at bias voltages of 1 and 5 V. Researchers should consider that light exposure significantly affects the photosensor parameters. As the light exposure time increases, the filling of deep traps, the generation of free carriers, and the devices’ charge accumulation capacity increase exponentially. These results support the characteristic current memory change described by eq 6. Clearly, the ZnO/1.0% Al/1.0% In sample exhibits superior properties compared with those of undoped ZnO for all calculated parameters. Longer exposure times reveal that the ZnO/1.0% Al/1.0% In sample exhibits more stable current memory, enabling more sensitive measurements, and that its photoconductive current gain increases significantly relative to that of ZnO. This indicates that the ZnO/1.0% Al/1.0% In sample can continue to be tested without being overwhelmed by noise, even as the optical signal increases.

**Table 4:** Photosensor Parameters of ZnO and ZnO/1.0% Al/1.0% In Devices as a Function of Bias Voltage and Exposure Time

sample	bias (V)	exposure time (s)	*S* (%)	*R* (A/W)	D* (×10^10^ Jones)	*G*
ZnO	1	5	4.20	0.05	0.93	0.15
		10	8.35	0.10	1.87	0.31
		30	21.35	0.28	4.96	0.86
ZnO/1.0% Al/1.0% In		5	5.67	0.13	1.78	0.42
		10	9.27	0.23	2.98	0.71
		30	23.63	0.63	7.86	1.95
ZnO	5	5	2.79	0.13	1.21	0.39
		10	4.61	0.21	2.01	0.66
		30	13.09	0.62	5.81	1.92
ZnO/1.0% Al/1.0% In		5	8.44	1.52	7.31	4.71
		10	12.29	2.36	11.0	7.32
		30	26.61	5.60	24.9	17.37

On the other hand, it is evident that the device operates over the voltage range of 1–5 V. However, when the bias voltage of undoped ZnO increases from 1 to 5 V, *S* decreases slightly whereas *R*, D*, and *G* increase modestly. This suggests that the 5 V bias approaches the operating limits of ZnO, and the device struggles to cope with increased heating and noise. The ZnO/1.0% Al/1.0% In sample, however, exhibits high sensitivity and robustness, even at 5 V, achieving a high photocurrent gain. This indicates that doping enables the device to operate effectively across a wide voltage range. For example, after exposure for 30 s, *R* and *G* increased approximately 8-fold and D* increased approximately 3-fold compared to the 1 V bias values, while the device sensitivity remained unaffected. The results show that the 1.0% Al:1.0% In dual-doping ratio is optimal and that the device effectively increases carrier density in deep traps. Furthermore, the device effectively manages the charge transfer between patterns under an increased electric field. On the other hand, it is evident that the device operates over the voltage range of 1–5 V. Figure 13c shows comparative changes in photosensor parameters at 1 and 5 V, plotted at the two ends of the same axis at 45° intervals in the radar plot. When the photosensor parameters at exposure times of 30, 10, and 5 s are examined, the 10 s exposure surpasses the 5 s exposure, whereas the 30 s exposure obscures the measurements for both 5 and 10 s. Its ability to operate at higher voltages enables a higher photocurrent and a clearer distinction from the dark current. High voltage endurance can increase the device’s resistance to structural degradation. The wide voltage range allows for a flexible response to various photocurrent conditions (or multilevel memory). Dual doping with 1.0% Al and In, which optimizes the defect levels and carrier concentration in the ZnO structure, increases the device’s potential not only as a sensor but also as a memory unit. The features mentioned here are demonstrated and supported by the device’s characteristic properties and by explanatory numerical and visual data shown in both Figure 13 and Table 4.

A schematic representation detailing the effect of double doping with Al and In on the system, current memory dynamics, energy band diagram, and carrier dynamics is given in Figure 14. The schematic energy band diagram was prepared specifically based on the PL spectrum shown in Figure 7b. Local lattice strain and structural disorder produced by Al and In ions in the ZnO lattice lead to the formation of tail states at the conduction band edge.^66^ Carriers ejected to these shallow levels (∼3.00–3.20 eV) by 400 nm (3.1 eV) excitation are transferred to deep trap centers (∼2.45–2.50 eV) via a nonradiative transition. The atomic structure around the trapped electrons in the deep traps undergoes lattice relaxation, shifting slightly to accommodate the electrons’ charge. This structural change creates a high activation energy barrier that prevents the carriers from returning to the valence band.^50^ At room temperature, the possibility of thermal detrapping of carriers from this barrier is suppressed. Experimental findings explain these mechanisms quite well. For example, the photocurrent of the ZnO/1.0% Al/1.0% In device begins to decrease after illumination for 60 s, but even after relaxation for 600 s (Figure 9), it can maintain a current memory level of ∼92% of the maximum current and ∼70% of the net photogeneration current. These physical mechanisms are also consistent with both the PL spectrum and electrical measurements.

**Figure 14:**
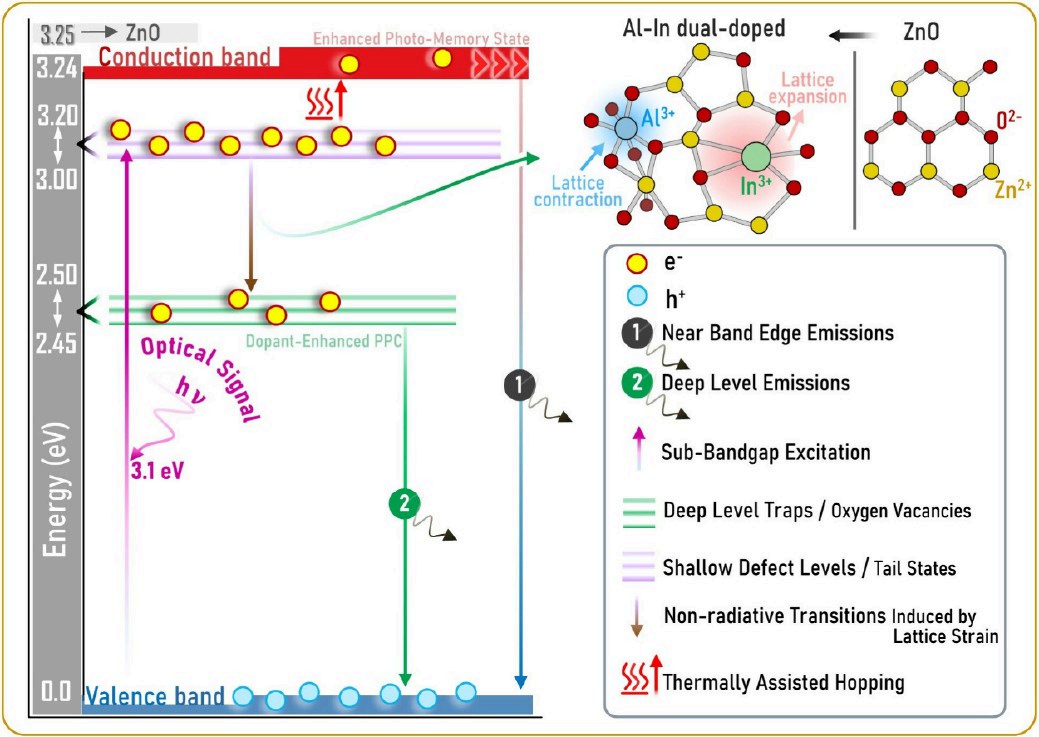
Schematic representation of the current memory dynamics basic mechanism and energy band diagrams of Al and In dual-doped ZnO-based photosensor devices.

## 4 Conclusions

In this study, the morphological, structural, optoelectronic, and memory dynamics performance of undoped, singly doped (Al or In), and dual-doped (Al and In) ZnO thin films synthesized by the SILAR method was systematically investigated. The results reveal that the doping ratio and dopant type significantly alter the crystal structure, surface morphology, and bond structure of ZnO. SEM analyses showed that undoped ZnO exhibited a uniform, dense, rod-like morphology, Al doping formed flake-like surface structures, In doping formed flower-like structures, and the particle thickness decreased from 400.1 to 201.6 nm. XRD results showed that all samples retained the hexagonal wurtzite structure; however, peak shifts and the decreased intensity of the (002) reflection were observed, and both changes were dependent on the dopant content. FTIR analyses and Raman spectra were preserved in all samples, but peaks were slightly shifted and broadened with Al and/or In doping. Here, the voltage and symmetry disturbances resulting from the replacement of Zn^2+^ ions with Al^3+^ and In^3+^ ions, and the increase in the intensity of the E_1_ (LO) mode (∼575–585 cm^–1^), demonstrate that the oxygen vacancy concentration and free-carrier density are increased by metal doping.

The changes observed in the PL (photoluminescence) spectra confirm the rearrangement of defects and energy levels in the structure. The local lattice voltage and structural disorder caused by Al and In ions in the ZnO lattice lead to the formation of tail states at the conduction band edge. Carriers thrown to shallow levels by sub-band gap excitation with 400 nm (3.1 eV) UV-A light reach deep trap centers via a nonradiative transition, forming the basic mechanism of the extended current memory. The ZnO/1.0% Al/1.0% In thin film device with IDE geometry exhibits a long decay time following UV irradiation and a superior ability to acquire and retain current information over successive cycles. With a low light intensity of 75 μW/cm^2^ and a minimum total optical dose of 4.5 mJ/cm^2^, the programmability of the ZnO/1.0% Al/1.0% In system demonstrates its high compatibility with energy-efficient photonic applications. The device exhibits strong current memory behavior, retaining 92.0% of its maximum current and approximately 70% of its net photogeneration current after excitation for 60 s followed by a 600 s decay period. Consistent with these measurements, the two-stage current decay observed in the devices after the UV light is switched off is described well by a dual-exponential fit model. The slow component of the exponential decay of photocurrent, τ_2_, is 302.9 s in undoped ZnO, whereas a long decay time of up to 533.3 s is obtained with the dual doping process using 1.0% Al and In. The presence of a structural barrier, supported by Raman and PL data, is thought to provide long-term data preservation by restricting the release of thermal energy carriers at room temperature. Bias tests in the range of 1–5 V showed that the ZnO/1.0% Al/1.0% In sample exhibited higher photosensitivity and photoresponsivity compared to those of pure ZnO at both low and high voltages. At a 5 V bias voltage, the sensing value in pure ZnO was approximately 1 × 10^10^ Jones, while in the ZnO/1.0% Al/1.0% In sample, this value increased to 2.5 × 10^11^ Jones. In 12 cycles of on–off tests, semiderivative analysis showed that the ZnO/1.0% Al/1.0% In sample exhibited approximately symmetrical current increase and decrease rates in each cycle, thus indicating effective filling and emptying of the traps. Dual doping with 1.0% Al and In, which optimizes the defect levels and carrier concentration in the ZnO structure, increases the device’s potential not only as a sensor but also as a memory unit.

Considering these results, dual doping with Al and In in ZnO provides an optimal balance among surface smoothness, crystal quality, and free-carrier density. These improvements enhance the potential of ZnO for photoresponsive, optoelectronic, sensor, and photonic applications, demonstrating that careful control of doping levels is critical to the design of functional thin films.

## Data Availability

Data will be made available upon request.
